# Pilot study to investigate differences in middle molecules, oxidative stress and markers of peripheral vascular disease in patients treated by high flux haemodialysis and haemodiafiltration

**DOI:** 10.1371/journal.pone.0258223

**Published:** 2021-10-06

**Authors:** Arkom Nongnuch, Chagriya Kitiyakara, Supawadee Sappadungsuk, Nuankanya Sathirapongsasuti, Kotcharat Vipattawat, Pin Zhang, Nathan Davies, Andrew Davenport

**Affiliations:** 1 Renal Unit, Department of Medicine, Faculty of Medicine, Ramathibodi Hospital, Mahidol University, Bangkok, Thailand; 2 Renal Unit, Department of Medicine, Faculty of Medicine, Chakri Naruebodindra Medical Institute, Ramathibodi Hospital, Mahidol University, Bangkok, Thailand; 3 Section of Translational Medicine, Faculty of Medicine Ramathibodi Hospital, Mahidol University, Bangkok, Thailand; 4 Bhumirajanagarindra Kidney Institute, Bangkok, Thailand; 5 Department of Medicine, University College London, London, United Kingdom; 6 UCL Centre for Nephrology, Royal Free Hospital, University College London, London, United Kingdom; Nazarbayev University School of Medicine, KAZAKHSTAN

## Abstract

**Background:**

Dialysis patients have an increased risk of mortality. Recently treatment with haemodiafiltration (HDF) has been reported to reduce mortality, particularly cardiovascular mortality, compared to standard high-flux haemodialysis (HD). However, why HDF may offer a survival advantage remains to be determined. So, we conducted a pilot study to explore differences in middle-molecules, inflammation and markers of vascular disease in patients treated by HD and HDF.

**Methods:**

Observational cross-sectional study measuring serum β2-microglobulin (β2M), Advanced Glycosylation End Products (AGEs) by skin autofluorescence (SAF), oxidative stress with ischaemia modified albumin ratio (IMAR) and peripheral vascular disease assessment using Ankle-Brachial Index (ABI), and arterial stiffness using Cardio-Ankle Vascular Index (CAVI).

**Results:**

We studied 196 patients, mean age 69.1 ± 12.4 years, 172 (87.8%) treated by HD and 24 (12.2%) by HDF. Age, body mass index, co-morbidity and dialysis vintage were not different between HD and HDF groups. Middle molecules; β_2_M (31±9.9 vs 31.2±10 ug/mL) and SAF (2.99±0.72 vs 3.0±0.84 AU), ABI (1.06±0.05 vs 1.07±0.10) and CAVI (9.34±1.55 vs 9.35±1.23) were not different, but IMAR was higher in the HD patients (38.4±14.8 vs 31.3 ± 17.4, P = 0.035)

**Conclusions:**

In this pilot observational study, we found patients treated by HDF had lower oxidative stress as measured by IMAR, with no differences in middle molecules. Lower oxidative stress would be expected to have diverse protective effects on the cardiovascular system Although we found no differences in ABI and CAVI, future studies are required to determine whether reduced oxidative stress translates into clinically relevant differences over time.

## Introduction

Although haemodialysis (HD) is life-sustaining for patients with chronic kidney disease (CKD) stage 5, mortality remains high, with an increased risk of cardiovascular disease (CVD) [1]. Patients are not only at risk of atheromatous coronary artery disease, but also arteriosclerosis, and as such HD patients have an increased risk for developing peripheral vascular disease (PVD) [2].

In clinical practice PVD can be assessed by measuring the ankle-brachial index (ABI) and arterial stiffness can be measured using the cardio ankle vascular index (CAVI). The ABI is the ratio of systolic blood pressure (SBP) between the ankle and brachial arteries, and the lower the ABI the greater the reduction in peripheral blood supply and risk of mortality [3]. Whereas the CAVI is based on arterial stiffness, and is theoretically independent of blood pressure [4]. The higher the CAVI, the stiffer the arterial tree, and as such an increased CAVI is now considered as one of the early markers of arteriosclerosis [5].

After adjusting for traditional risk factors including age, gender, smoking history and lipid profiles, then inflammation is reported to be a significant prognostic factor associated with PVD in patients with CKD [6]. Although C reactive protein (CRP) is a marker of inflammation, CRP has a very short half-life and as such may have greater day to day variability. Advanced glycosylation end products (AGEs) are middle sized molecules which are deposited in the skin, and this can be measured by autofluorescence. Skin autofluorescence provides a more chronic assessment of AGEs as there is some clearance during a dialysis session with high-flux-HD and HDF, so serum AGEs can vary with the dialysis schedule, and timing of blood sampling. Higher skin autofluorescence (SAF) is associated with greater mortality for HD patients [7]. Oxidative stress leads to a structural change in the albumin molecule, and the amount of oxidative damage can be determined by measuring ischaemia modified albumin (IMA). Observational studies have reported that increased IMA is associated with greater mortality in many populations, including HD patients [8, 9].

Haemodiafiltration (HDF) is gaining popularity in Europe, and an individual patient analysis of four recent major clinical trials reported that all-cause survival was greater for dialysis patients treated with HDF compared to treatment with standard high-flux HD [10], with a particular reduction in cardiovascular mortality [11]. However, the basis as to why HDF may provide a survival advantage over HD remains to be determined. We therefore conducted a pilot study to determine whether there were differences in middle molecules (β_2_ microglobulin and SAF), IMA, assessments of PVD and arterial stiffness in dialysis patients treated by high-flux HD and HDF.

## Materials and methods

**Fig 1:**
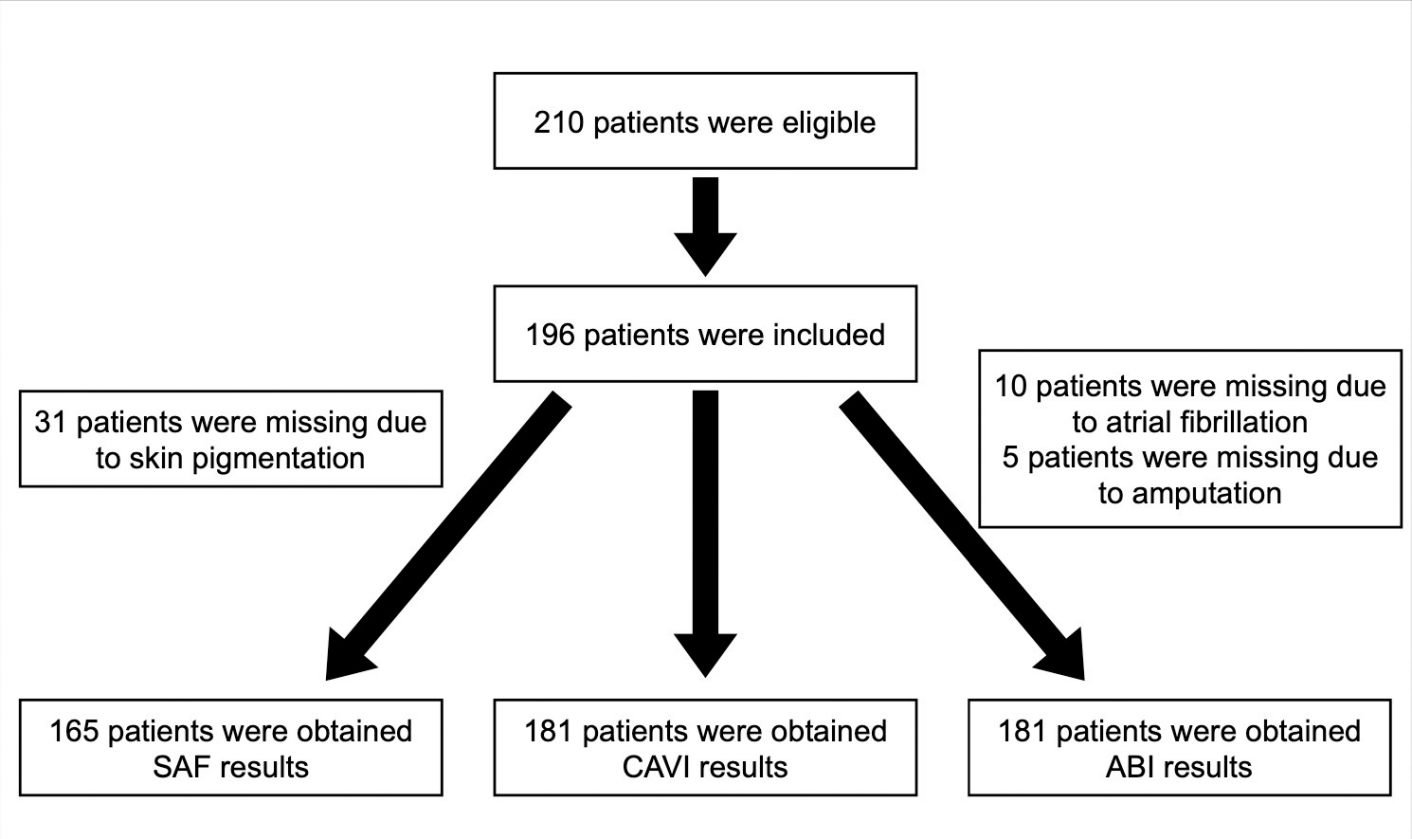
Study consort patient flow diagram.

We enrolled HD patients dialysing in 3 centres in the central Bangkok region in our pilot observational study between November 2015 and March 2016 (Fig 1). Medical histories and results of routine blood results, including high sensitivity CRP (hsCRP) and beta-2 microglobulin (β_2_M), were obtained from hospital records. All patients dialysed 2 or 3 times a week, for a minimum of 4 hours per session. The target dialysis session Kt/V was >1.2 for thrice weekly HD and >2.0 for twice weekly HD. This was designed so that patients with different dialysis schedules would receive the same weekly equilibrated dialyser clearance (Kt/V). If patients failed to achieve the sessional target Kt/V with 4 hours, then the dialysis session time was increased appropriately. For those patients treated by HDF, then the target for convective clearance was more than 20 L per session, and again if required dialysis session times were extended to ensure targets were achieved. All HD and HDF treatments used a high flux dialyser and ultrapure quality dialysis water. The decision as to whether patients had been treated by HD or HDF was at the discretion of the supervising renal clinician. All patients had been treated by either high-flux HD or HDF for a minimum of three months prior to study entry, and within that period no HDF patient had been treated by high-flux HD, and vice-versa.

### The ischaemia modified albumin ratio (IMAR) measurement

Albumin is structurally altered by oxidative stress and then is unable to bind cobalt at the metal binding site. By adding cobalt, then cobalt not bound by albumin can be detected by a yellow/brown colourimetric reaction between free cobalt and dithiothreitol [8]. We tested 30ul plasma samples in duplicate and standards in triplicate in a 96 well flat-bottomed plate with 85ul 3-(N-morpholino) propanesulfonic acid buffer (20 mM) and 60ul cobalt chloride solution (840 uM). After mixing incubation at 22°C for 10 minutes., 25 ul dithiothreitol (DTT)(2 mM) was then added into each well and mixed vigorously for 2 minutes. Absorbance was read at 470 nm and 650 nm (FLUO star Omega, Germany) for quantification. A second plate was prepared, using 25ul distilled water instead of DTT. The final absorbance was calculated comparing plates with and without DTT. Albumin concentrations were measured by the bromocresol purple method, and the proportion of IMA calculated as the inverse of free cobalt measured, and then expressed as a ratio (IMAR) to a serum albumin concentration of 44 g/L [9].

### The skin autofluorescence (SAF) measurements for advanced glycosylation end products (AGEs) estimation

SAF measurements were made using an AGE reader (DiagnOptics, Groningen, The Netherlands), which uses ultraviolet light range 300–420 nm for excitation of AGEs deposited in subcutaneous tissues and then measures the reflected autofluorescence light by spectrophotometer [12]. SAF was measured in the non-fistula forearm, at room temperature in a semi-dark room environment and the average of three measurements recorded in arbitrary units (AU). The inter-observer and intra-observer variability of SAF measurement was approximately 5% [13]. 24-hour urine collections are unreliable in dialysis patients, due to changes in urine volume and solute clearances varying with the time from the previous dialysis session, so we used β2M and SAF as surrogate markers of dialysis clearance and residual renal function.

### The Cardio Ankle Vascular Index (CAVI) and Ankle-Brachial Index (ABI) measurements

CAVI and ABI were measured using the vascular screening system (VaSera VS-2000, Fukuda Denshi, Japan). Four cuffs were placed on the limbs and CAVI measured as to the difference in the pulse wave velocity arriving to the ankles relative to the heart [14]. All measurements were made at room temperature, and after patients had rested and abstained from smoking or eating for at least 2 hours. The inter-observer and intra-observer variability of CAVI measurement were reported to be around 3% [15]. ABI was measured in supine position using pneumatic-cuffs placed over the brachial artery and ankle, and the pressure of brachial, dorsalis pedis and posterior tibial arteries measured using a hand-held continuous wave Doppler probe (5–10 MHz), with a reported measurement variability of 4% [16].

### Statistical analysis

Normally distributed numeric data are presented as mean and standard deviation and non-parametric data as median and interquartile range. The student t test or Mann-Whitney U-tests were used to compare continuous variables for analysing parametric and non-parametric variables respectively, with appropriate post-hoc testing. Categorical data is presented as number (%) and Chi-squared or Fisher’s exact test were used to analyse categorical variables, with appropriate adjustments for small numbers. Factors associated with IMAR were analysed initially in a univariate linear regression model and those variables with a p value < 0.1 were then included in multivariate linear regression model (SPSS 25 IBM Armonk, New York, USA). Variables which were not normally distributed were log transformed prior to inclusion. Statistical significance was taken with a p value <0.05. The graph was plotted using R statistic (www.r.project.org).

### Ethics

This study was approved by the local ethics committee (Ramathimodi Hospital, Mahidol University MURA 2015/728) and conducted according to the principles of the declaration of Helsinki, with patients providing written informed consent, and registered with the Thai Clinical Trials Registry (TCTR20200306001).

## Results

**Fig 2:**
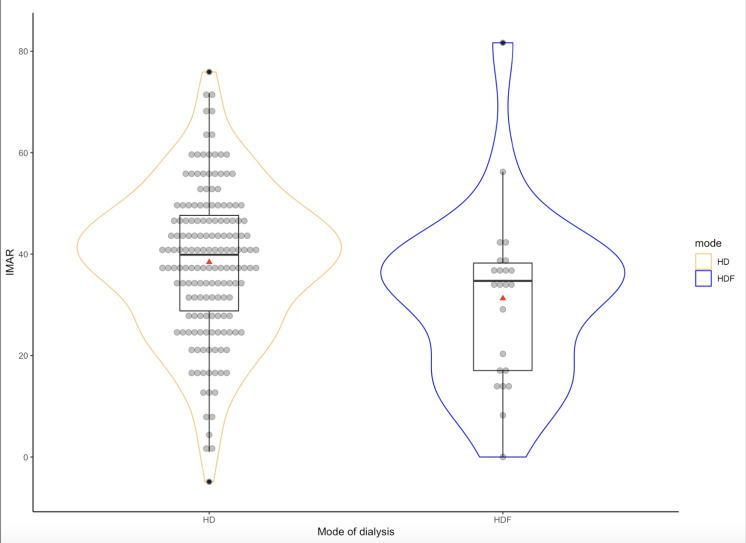
Ischaemia modified albumin ratio (IMAR) in patients treated by high-flux haemodialysis (HD) and haemodiafiltration (HDF). Red triangular symbols represent mean for treatment group (P = 0.035) and the horizontal black line represents the median for treatment group (P = 0.013).

210 HD patients were eligible for study, but 14 patients declined to participate (Fig 1). We therefore enrolled 196 patients, mean age 69.1 ± 12.4 years, 172 (88%) were treated by high-flux HD and 24 by HDF. In this pilot study we wished to determine whether there were differences with HDF treatment (Table 1). More women were treated by HDF, and more were smokers. However, smoking was defined as any use of cigarettes, so included the occasional cigarette to regular users smoking 0.25–0.5 packets/day. Assessment of PVD showed no differences in CAVI or ABI (Table 1), and there was a similar percentage of patients with an increased CAVI of >9.0 (HD 58.9 vs HDF 65.2%) and a reduced ABI of <0.9% (HD 84.8 vs HDF 87%). There were no differences in middle molecules, assessed by serum β_2_M or SAF. IMAR for the whole group was 37.5±15.2, and IMAR was significantly lower in the HDF group (Fig 2).

**Table 1:** Patient demographics, relevant medical conditions laboratory investigations, and assessment or peripheral vascular disease and comparison of treatment with high-flux haemodialysis (HD) and haemodiafiltration.

Parameter	All patients (n = 196)	HD (n = 172)	HDF (n = 24)	p value
Age, years	69.5±13.1	69.2±13.17	69.0±13.3	0.94
Female gender, %	52.1	46	100	<0.001
Body mass index, kg/m^2^	22.1±4.8	22.04±4.73	23.24±5.23	0.25
Dialysis vintage months, (IQR)	19.1 (21.5)	20.1 (21.6)	16.7 (20.1)	0.38
3 x week dialysis, %	48	47	58	0.30
Serum albumin, g/L	37.1±4.0	37.2 ±3.9	36.3±3.9	0.27
Haemoglobin, g/dL	11.0±1.4	11.06±1.41	10.85±1.48	0.50
Glycated haemoglobin, %	6.1±1.3	6.10±1.29	6.10±1.34	0.92
Serum calcium, mg/dL	9.2±0.7	9.21±0.68	9.08±0.82	0.36
Serum phosphate, mg/dL	4.6±1.4	4.58±1.39	4.39±1.50	0.53
Parathyroid hormone, pg/mL	364 (445)	391 (455)	296 (367)	0.33
B_2_Microglobulin, ug/mL	31.0±9.9	31.2±10.0	30.7±8.3	0.81
High sensitivity C reactive protein, mg/L	0.15 (0.47)	0.15 (0.38)	0.21 (0.64)	0.56
Skin autofluorescence, AU	2.99±0.72	3.03±0.84	3.18±0.62	0.42
Cardio ankle vascular index	9.34±1.55	9.34±1.60	9.35±1.23	0.99
Cardio ankle vascular index>9, %	9.3	10.1	4.1	0.35
Ankle-brachial index	1.06±0.15	1.06±0.15	1.07±0.16	0.79
Ankle-brachial index <0.9, %	14.9	15.2	13.0	0.79
Diabetic, %	57.1	56.4	62.5	0.57
Smoker, %	21	18	42	0.01
Coronary artery disease, %	32.7	33.1	33.3	0.98
Coronary artery bypass, %	25.4	23.9	16.6	0.28
Previous Stroke, %	9.3	10.1	4.1	0.35
Hypertension, %	94	93	100	0.18

(HDF) Results expressed as mean ±standard deviation, or median (interquartile range). P values comparing HD and HDF.

To investigate factors associated with IMAR we constructed a univariate linear regression model (Table 2), and we noted lower IMAR with HDF, and also an association with CAVI, and serum calcium, although this association may be confounded by calcium binding to albumin, and both calcium and IMAR being adjusted for serum albumin. In a multivariate model only HDF remained associated with a lower IMAR (odds ratio 0.36, 95% confidence limits 0.14 to 0.94, p = 0.037).

**Table 2:** Univariate analysis of factors associated with ischaemia modified albumin ratio (IMAR).

Parameters	Β coefficient	95% confidence limits	P value
Age years	-0.016	0.19 to 0.15	0.85
Female gender	-0.29	-4.5 to 4.4	0.99
Body mass index	0.07	-0.4 to 0.5	0.77
haemodiafiltration	-7.14	-13.8 to -0.51	0.035
Dialysis session frequency 3 x vs 2 x week	-0.003	-4.4 to 4.4	0.99
Skin autofluorescence	2.46	-0.54 to 5.4	0.11
Log cardio ankle vascular index	-31.4	-62.2 to -0.6	0.046
Increased cardio ankle vascular index >9	-6.51	-11.0 to -2.0	0.005
Log ankle brachial index	-27.28	-60.2 to 5.7	0.10
Normal ankle brachial index	-1.25	-7.5 to 5.1	0.70
Serum albumin	15.01	9.9 to 20.1	0.0001
haemoglobin	1.26	-0.3 to 2.8	0.12
Glycated haemoglobin	-0.75	-2.4 to 0.9	0.38
Serum calcium	4.77	1.7 to 7.9	0.003
Serum phosphate	-0.41	-2.0 to 1.2	0.61
Serum Parathyroid hormone	0.001	-2.99 to 8.05	0.466
serum β2 microglobulin	0.12	-0.13 to 0.36	0.36
Log high sensitivity C reactive protein	-2.5	-6.8 to 1.8	0.25
Smoker	-2.9	-8.2 to 2.3	0.26
Diabetes	2.29	-2.2 to 6.7	0.31
Hypertension	0.78	-8.6 to 10.1	0.17
History of coronary artery disease	-2.07	-6.8 to 2.6	0.39
History of coronary artery bypass surgery	-1.71	-6.8 to 3.4	0.51
History of stroke	3.52	-4.2 to 11.2	0.37

## Discussion

Treatment with HDF was been suggested to reduce mortality in dialysis patients compared to standard high-flux HD [9], but the mechanism as to why HDF may improve survival remains to be determined. One potential explanation would be that HDF increases middle molecule clearances, and as such we conducted a pilot study first measuring middle molecules, but found no difference in serum β_2_M. Maduell and colleagues also reported no differences in serum β_2_M between HDF and high-flux HD groups in their study reporting higher survival with HDF [17]. This failure to demonstrate a difference in serum β_2_M could have been due to differences in residual renal function. However, in Thailand, clinical practice is to generally initiate dialysis treatments much later than in many Western European countries, due to differences in pre-dialysis care. So the number of patients who start dialysis with some residual renal function is much less, and as such we did not request formal measurements of residual renal function. β_2_M is a middle sized molecule, and so clearance is increased with longer haemodialysis session times, and as such we may achieved greater β_2_M clearance with our practice of increasing HD session times to more than 4 hours to achieve kt/V targets.

We also measured tissue AGEs as a more chronic measurement of middle molecule accumulation using skin autofluorescence, as increased SAF has been reported to be associated with increased mortality in HD patients [18]. However, we observed no difference in SAF between HDF and high-flux HD groups. However, AGEs can be affected by factors other than dialysis clearance [19], including diet [20], and patients in Thailand may potentially have a different diet compared to patients in Europe [21].

Others have suggested that HDF reduces cardiovascular mortality [11], and as vascular stiffness measured by pulse wave velocity is reported to be a major risk factor for cardiovascular mortality in HD patients [22], we measured CAVI and ABI. However, we found no differences between groups, although more of the HDF group were smokers, and smoking is a recognised risk factor for PVD. Although smoking is reported to increase both vascular stiffness and pulse wave velocity in the general population [23], studies in dialysis patients have failed to confirm this association [24]. Firstly, the cigarrete usage in our patients was much lower than that reported in many studies, and secondly vascular stiffness in dialysis patients may be more affected by deposition of calcium and sodium in the media of major arteries compared to the general population [24].However, our pilot observational study only made a single time point measurement of CAVI and ABI, and although treatment time was not dissimilar between groups, changes in vascular stiffness may take a much longer time before any changes due to dialysis modality become evident [25], and similarly we have no information about pre-dialysis care and blood pressure control.

Oxidative stress is reported to be increased in patients with CKD and HD patients [26, 27]. As oxidative stress is associated with both increased cardiovascular and peripheral vascular disease [8], we measured ischaemia modified albumin and calculated the IMAR to allow comparison between patients. Despite an increased number of smokers in the HDF group, IMAR was significantly lower in the HDF group, and remained independently associated with HDF in a multivariable model. Our results would support two earlier small observational studies. One in children, which reported lower oxidised low-density lipoprotein with HDF [26], and a second study based on 38 patients from Japan which reported that treatment with pre-dilution haemodiafiltration resulted in a higher mercapto-albumin to albumin ratio [27].However, not all studies have observed changes in oxidative stress, with one small study reporting varying results, with some lowering of total antioxidant capacity, but no change in superoxide dismutase [28]. Although slightly more HDF patients received were treated by thrice weekly schedules, haemodialysis treatments have themselves been reported to generate oxidative stress and generate peroxynitrite-induced oxidation of plasma lipids [29].

Our present pilot study has several limitations. Firstly, as we only have cross-sectional data, we can only demonstrate an association between HDF and a reduction in IMAR, rather than attribute a causality. In our study all patients had been treated for a minimum of three months of either high-flux HD or HDF, with no crossing over of treatment modes, and before that only around 10% of patients had changed from HD to HDF. Although we would suggest that this was sufficient time to expect changes in β_2_M, SAF and IMAR, it may not have been of sufficient time to establish differences in ABI or CAVI. As such, we cannot comment on whether measurements of CAVI and ABI in particular or SAF and IMAR change over time with longer exposure to HDF treatments compared to high-flux HD. Secondly, we do not have direct measurements of residual renal function. As the common practice in Thailand, most patients starting HD have little residual renal function than European countries. We used serum β_2_M and tissue AGE deposition, as surrogates of combined dialysis and residual renal clearances, but as these were similar for HD and HDF patients it is unlikely that major differences in dialysis clearance and residual renal function could account for the differences we found in IMAR. Thirdly, as this was an observational study then the assignment of treatment to HDF was not random, we cannot exclude a selection bias, in this pilot study, however age and co-morbidity were not different, but as β_2_M and SAF were similar between treatment groups, then this would suggest that he difference in IMAR was associated with HDF treatments. Oxidative stress has been reported to be lower in women, but only those of reproductive age, and not in post-menopausal women [30]. As all our female patients treated by HDF were post-menopausal, with an average age of 69 years, we do not think that gender alone could account for our IMAR results, and gender was not retained in the multivariable model.

## Conclusions

We report in this observational pilot study that HDF treatment is associated with lower oxidative stress as measure by IMAR. Oxidative stress underpins the increased atherosclerosis, endothelial dysfunction and cardiac injury observed in kidney dialysis patients. The benefits of HDF to slow atherosclerosis and endothelial dysfunction in ESRD are remaining elucidated. Thus, larger and well controlled studies are required to explore whether this is a causal relationship, and importantly whether a reduction in oxidative stress accounts for clinically relevant differences between dialysis modalities.

## Supporting information

S1 Checklist STROBE statement—checklist of items that should be included in reports of *cross-sectional studies*. (DOCX)

S1 Fig SAF in patients treated by high-flux haemodialysis (HD) and haemodiafiltration (HDF). Red triangular symbols represent mean for treatment group (P = 0.42).
(PNG)

S2 Fig CAVI in patients treated by high-flux haemodialysis (HD) and haemodiafiltration (HDF). Red triangular symbols represent mean for treatment group (P = 0.99).
(PNG)

S3 Fig ABI in patients treated by high-flux haemodialysis (HD) and haemodiafiltration (HDF). Red triangular symbols represent mean for treatment group (P = 0.79).
(PNG)

S1 Data (XLSX)
