# Supplementary figures and images for: Pilot study to investigate differences in middle molecules, oxidative stress and markers of peripheral vascular disease in patients treated by high flux haemodialysis and haemodiafiltration
Source: PLoS One. 2021 Oct 6;16(10):e0258223. doi: 10.1371/journal.pone.0258223 (PMC8494338; doi:10.1371/journal.pone.0258223)

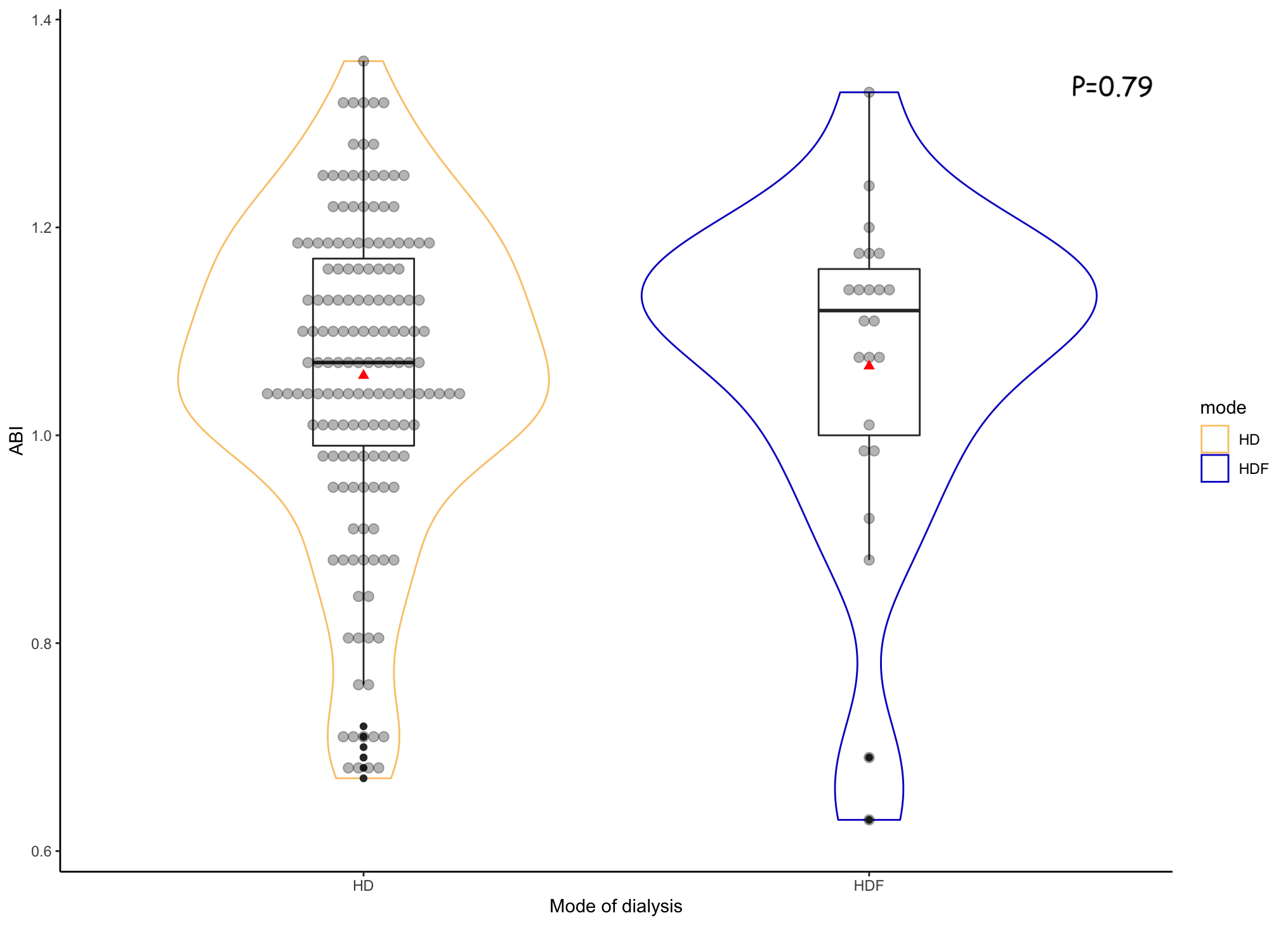

Supplement: S1 Fig — Red triangular symbols represent mean for treatment group (P = 0.42). (PNG) [file pone.0258223.s002.png]

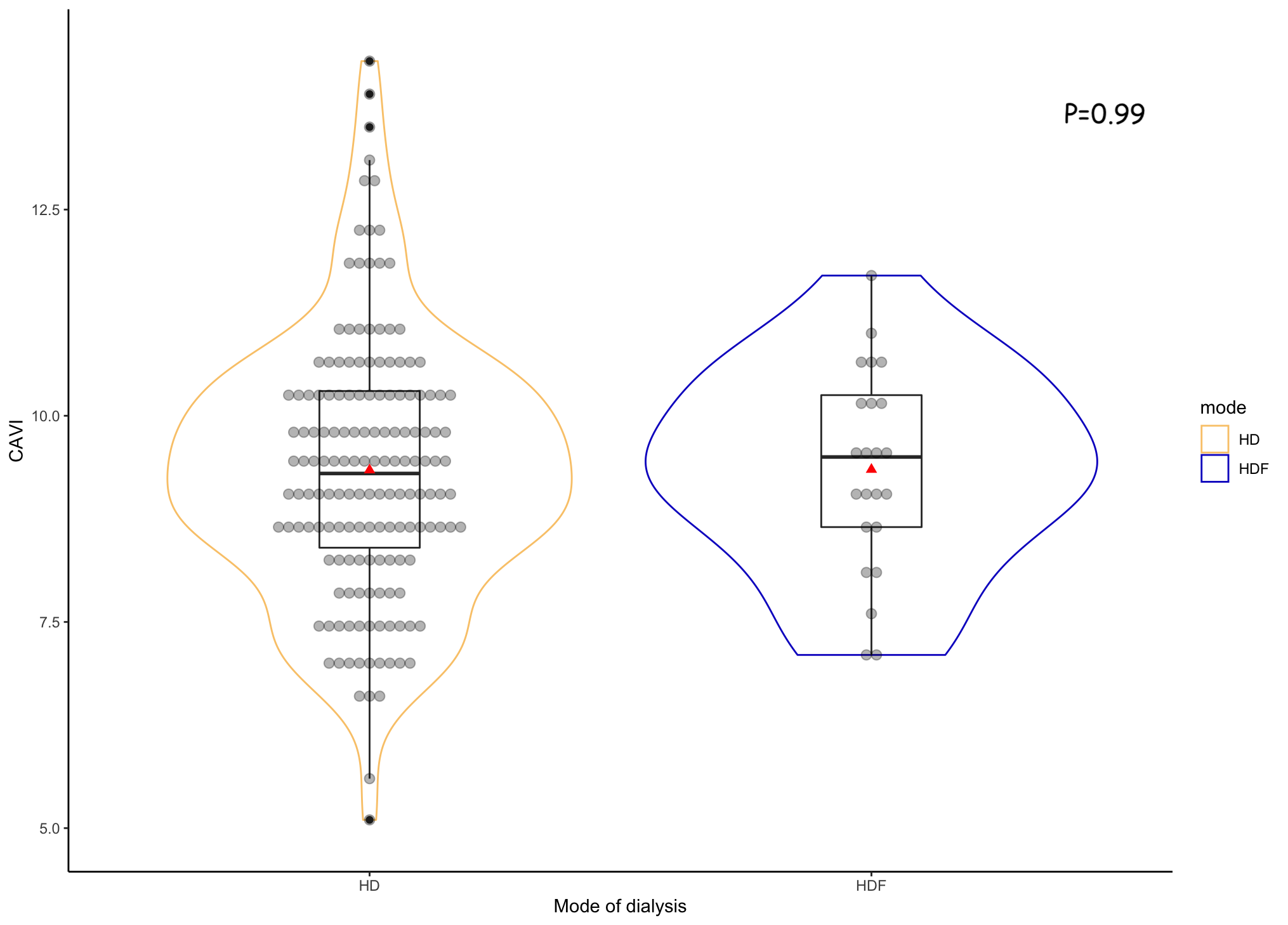

Supplement: S2 Fig — Red triangular symbols represent mean for treatment group (P = 0.99). (PNG) [file pone.0258223.s003.png]

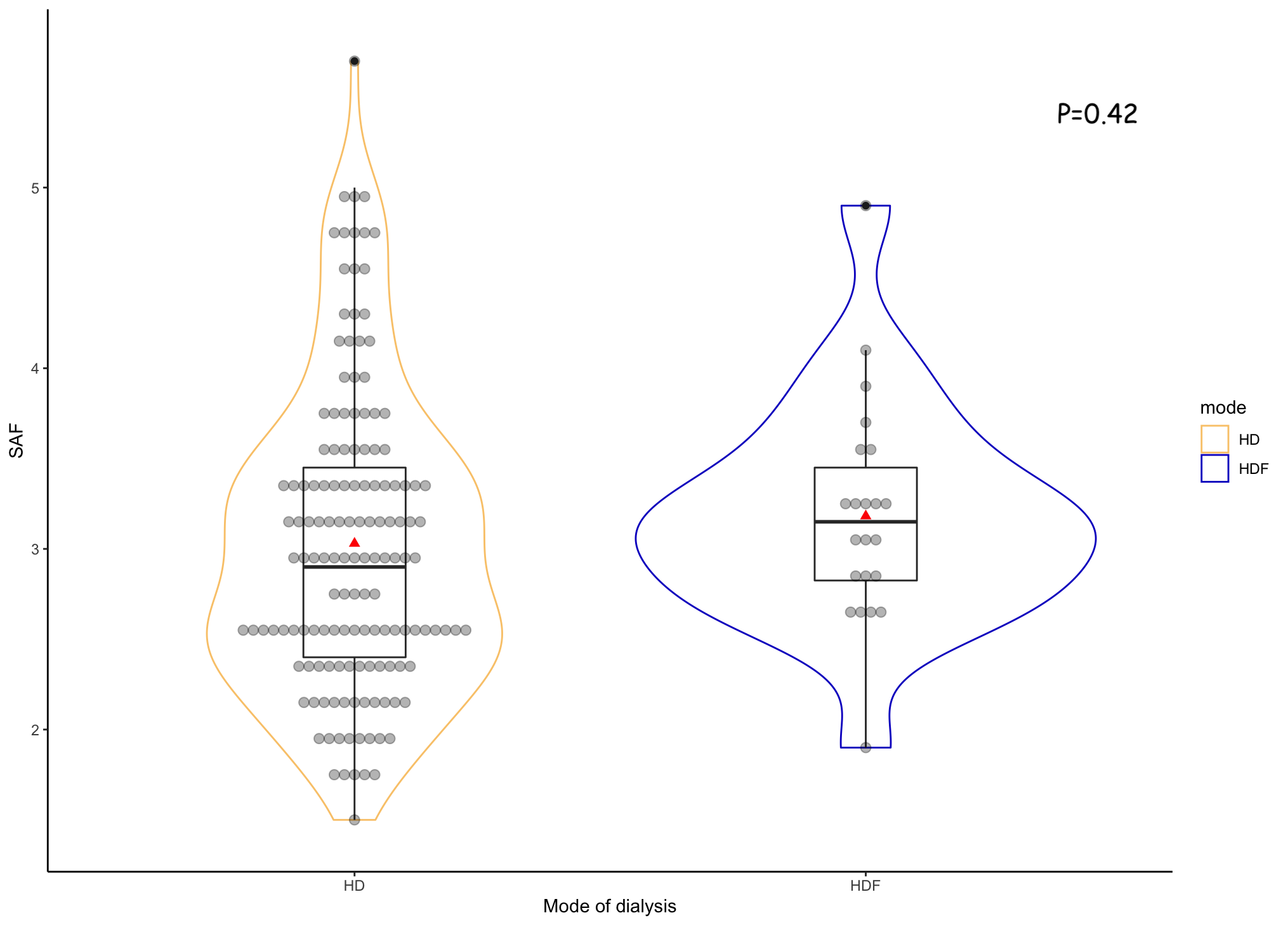

Supplement: S3 Fig — Red triangular symbols represent mean for treatment group (P = 0.79). (PNG) [file pone.0258223.s004.png]
